# Clinical Characteristics of Q Fever and Etiology of Community-Acquired Pneumonia in a Tropical Region of Southern Taiwan: A Prospective Observational Study

**DOI:** 10.1371/journal.pone.0102808

**Published:** 2014-07-17

**Authors:** Chung-Hsu Lai, Lin-Li Chang, Jiun-Nong Lin, Wei-Fang Chen, Yu-Feng Wei, Chien-Tung Chiu, Jiun-Ting Wu, Chi-Kuei Hsu, Jung-Yueh Chen, Ho-Sheng Lee, Hsi-Hsun Lin, Yen-Hsu Chen

**Affiliations:** 1 Graduate Institute of Medicine, College of Medicine, Kaohsiung Medical University, Kaohsiung City, Taiwan; 2 Division of Infectious Diseases, Department of Internal Medicine, E-Da Hospital/I-Shou University, Kaohsiung City, Taiwan; 3 Division of Infection Control Laboratory, E-Da Hospital/I-Shou University, Kaohsiung City, Taiwan; 4 Faculty of Medicine, Department of Microbiology, College of Medicine, Kaohsiung Medical University, Kaohsiung City, Taiwan; 5 Division of Pulmonary Medicine, Department of Internal Medicine, E-Da Hospital/I-Shou University, Kaohsiung County, Taiwan; 6 Institute of Clinical Medicine, National Yang-Ming University, Taipei City, Taiwan; 7 Division of Infectious Diseases, Department of Internal Medicine, Kaohsiung Medical University Hospital, Kaohsiung Medical University, Kaohsiung City, Taiwan; 8 School of Medicine, College of Medicine, Kaohsiung Medical University, Kaohsiung City, Taiwan; University of Arkansas for Medical Sciences, United States of America

## Abstract

**Background:**

The clinical characteristics of Q fever are poorly identified in the tropics. Fever with pneumonia or hepatitis are the dominant presentations of acute Q fever, which exhibits geographic variability. In southern Taiwan, which is located in a tropical region, the role of Q fever in community-acquired pneumonia (CAP) has never been investigated.

**Methodology/Principal Findings:**

During the study period, May 2012 to April 2013, 166 cases of adult CAP and 15 cases of acute Q fever were prospectively investigated. Cultures of clinical specimens, urine antigen tests for *Streptococcus pneumoniae* and *Legionella pneumophila*, and paired serologic assessments for *Mycoplasma pneumoniae*, *Chlamydophila pneumoniae*, and Q fever (*Coxiella burnetii*) were used for identifying pathogens associated with CAP. From April 2004 to April 2013 (the pre-study period), 122 cases of acute Q fever were also included retrospectively for analysis. The geographic distribution of Q fever and CAP cases was similar. Q fever cases were identified in warmer seasons and younger ages than CAP. Based on multivariate analysis, male gender, chills, thrombocytopenia, and elevated liver enzymes were independent characteristics associated with Q fever. In patients with Q fever, 95% and 13.5% of cases presented with hepatitis and pneumonia, respectively. Twelve (7.2%) cases of CAP were seropositive for *C. burnetii* antibodies, but none of them had acute Q fever. Among CAP cases, 22.9% had a CURB-65 score ≧2, and 45.8% had identifiable pathogens. *Haemophilus parainfluenzae* (14.5%), *S. pneumoniae* (6.6%), *Pseudomonas aeruginosa* (4.8%), and *Klebsiella pneumoniae* (3.0%) were the most common pathogens identified by cultures or urine antigen tests. Moreover, *M. pneumoniae*, *C. pneumoniae*, and co-infection with 2 pathogens accounted for 9.0%, 7.8%, and 1.8%, respectively.

**Conclusions:**

In southern Taiwan, Q fever is an endemic disease with hepatitis as the major presentation and is not a common etiology of CAP.

## Introduction

Q fever is a zoonosis caused by infection of *Coxiella burnetii*, an obligate intracellular microorganism [1]. Cattle, sheep, and goats are the major animal reservoirs and most human infection results from inhalation of aerosolized particles contaminated with the organism shed from infected animals, particularly in the reproductive tract material, dairy products, feces, and urine. Ingestion of contaminated raw milk is another, less common route [2]. In acute Q fever, which develops after the primary infection, nearly 60% of patients are asymptomatic, and the remainder may present with fever and influenza-like symptoms accompanied with varying degrees of pneumonia or hepatitis [3]. Chronic Q fever may occur months to years after the primary infection. Infective endocarditis is the most common complication, particularly in immunocompromised patients, pregnant patients, or those with cardiovascular abnormalities [3]. With regard to symptomatic acute Q fever, a geographic difference is found between the two major clinical presentations with pneumonia predominant in Nova Scotia, Canada [4], Switzerland [1], the north of Spain [5], and the Netherlands [6], [7] and hepatitis predominant in France [8], the south of Spain [9], California [10], Ontario, Canada [11], and Taiwan [12].

Despite the worldwide distribution of human Q fever, it is mostly reported in countries located in temperate zones (between 23.5°N and 66.5°N, and 23.5°S and 66.5°S), including Europe (i.e., France, the Netherlands, Spanish, Germany, Bulgaria, Spain, and Switzerland) [1], [5], [6], [8], [9], [13], North America (i.e., USA and Canada) [4], [10], [11], [14], Northeast Asia (i.e., Japan and South Korea) [15], [16], Western Asia (i.e., Israel) [17], Northwest Africa (i.e., Spain’s Canary Islands) [18], and South Australia [19]. In countries located in tropical zones (between 23.5° north and south of the equator), however, Q fever is rarely reported. These countries include North Australia [20], French Guiana (located at north coast of South America) [21], Taiwan [22], Thailand [23], Senegal and Togo (located at West Africa) [24], [25], and Tanzania and Kenya (located at East Africa) [26], [27]. Many of the studies from these regions are serological surveys that do not clarify the clinical characteristics.

Q fever is an important etiological agent of community-acquired pneumonia (CAP), particularly so-called atypical pneumonia [28]. However, Q fever is not universally included in the etiologic investigation of CAP. In western countries, Q fever accounts for 0.4–10% of CAP [29]–[37]. In endemic regions, the percentage of Q fever in CAP is reported to be as high as 20.1% in Spain’s Canary Islands [18] and 24.4% in French Guiana [21]. In Asia, Q fever in pneumonia is rarely investigated [38]–[40], and it accounts for 4.2% of CAP in Japan [38], 1.2% in Korea [39], and 0.4% in Hong Kong [40].

Previous retrospective studies revealed that hepatitis is the predominant presentation of acute Q fever in Taiwan [12], [22]. However, a selection bias might exist in which clinicians tend to order Q fever tests in febrile patients who present with hepatitis when no pneumonia is found on the chest-x ray. We had previously reported that up to 60% and 25% of acute Q fever cases had seropositivity of *Mycoplasma pneumoniae* IgM and *Chlamydophila pneumoniae* IgM, respectively, by the examination of enzyme-linked immunosorbent assays (ELISA) [41]. This observation suggested the possibility of an overestimation of the prevalence of atypical pneumonia and an underestimation of the true prevalence of Q fever pneumonia. To the best of our knowledge, only two prospective studies of the etiology of CAP have been reported from Taiwan; however, Q fever was not included in the survey [42], [43].

The aim of this study is to investigate the clinical characteristics of Q fever and the etiology of CAP in southern Taiwan, located in the tropical zone.

## Methods

### Patient eligibility and recruitment criteria

#### Inclusion criteria

Patients who are aged ≧18 years old and admitted to a pulmonary medicine ward with the diagnosis of pneumonia are prospectively evaluated for recruitment into the study. Pneumonia is defined as the presence of a new infiltrate on a chest-x ray plus at least one of the following: fever (body temperature ≥38.0°C) or hypothermia (body temperature ≤35.0°C), new cough with or without sputum production, pleuritic chest pain, dyspnea, or altered breath sounds on auscultation [44]. The definition of CAP is pneumonia which is excluded from the diagnostic criteria of hospital-acquired pneumonia (HAP), ventilator-associated pneumonia (VAP), and healthcare-associated pneumonia (HCAP) [45].

#### Exclusion criteria

Patients who do not have CAP. To avoid the recruitment of patients who might be colonized by or have a repeated infection with specific pathogens due to underlying pulmonary disorders, those who have pulmonary malignancy, bronchiectasis, tracheostomy, and active or newly diagnosed tuberculosis are excluded. To involve the patients who are vulnerable to contract Q fever, patients are excluded if they are in non-ambulatory condition before evaluation. Those who refused recruitment into the study after explanation are also excluded.

### Clinical specimen collection, disease severity, and treatment of CAP

Cultures of available respiratory tract specimens and blood are performed according to standard clinical practice and procedures. A pair of sera including acute (≦14 days from the disease onset) and convalescent phase (>14 days from the disease onset and at least 7 days apart from the date of acute phase specimen) samples are collected for serological diagnosis of Q fever, mycoplasma pneumonia, and chlamydophila pneumonia. Urine is collected for *Streptococcus pneumoniae* and *Legionella pneumophila* antigen tests immediately after the patient is included. A CURB-65 score is used for evaluating the severity of CAP [46]. Because this is a prospective observational study, the treatment of CAP is fully dependent on the doctors of the patients, and no intervention is conducted.

### Identification of specific causative pathogens of pneumonia

*Streptococcus pneumoniae* infection is defined by the isolation of *S. pneumoniae* from blood or respiratory tract specimens or a positive result on a urine antigen test for *S. pneumoniae* (Alere BinaxNOW *Streptococcus pneumoniae* Antigen Card). *M. pneumoniae* and *C. pneumoniae* infection is defined by the seroconversion of IgG or a positive result of an IgM test in paired sera for each disease by commercial ELISA kits (*M. pneumoniae* IgG [SeroMP™ IgG]/IgM [SeroMP™ IgM] and *C. pneumoniae* IgG [SeroCP™ IgG]/IgM [SeroCP™ IgM]) (Savyon Diagnostics, Ashdod, Israel). *Legionella pneumophila* infection is defined by a positive result on a urine antigen test for *L. pneumophila* (Alere BinaxNOW *Legionella* Urinary Antigen Card).

### Diagnosis of acute Q fever

Q fever is a notifiable disease in Taiwan, and paired blood specimens of clinically suspected cases are sent to the Centers for Disease Control of Taiwan (Taiwan CDC) for a confirmatory test. Acute Q fever was diagnosed by an anti-phase II antigen IgG titer of ≥1∶320 and an anti-phase II antigen IgM titer of ≥1∶80, a four-fold or greater increase of anti-phase II antigen IgG titer in paired sera, or a positive blood test for *C. burnetii* DNA, detected by polymerase chain reaction (PCR) [47]. Immunofluorescence assay kits (Q fever IFA [IF0200G] and [IF0200M], Focus Diagnostics) were used for the determination of antibodies against *C. burnetii* in CAP cases, and the kits were the same as those used by the Taiwan CDC. Q fever hepatitis is defined as acute Q fever presents with an elevated values of liver enzymes (glutamate-pyruvate transaminase [GPT]>44 U/L or glutamate-oxaloacetate transaminase [GOT]>38 U/L). Q fever pneumonia indicates acute Q fever fulfills the definition of pneumonia used for CAP.

### Data collection and geographic distribution of cases

#### Study period (May 2012 to April 2013)

Data from enrolled CAP cases and acute Q fever diagnosed during the study period were recorded prospectively; the data included demographic information, clinical characteristics, results of laboratory and imaging examinations, and treatment response. The risk factors regarding the acquisition of Q fever included occupation, a recent history of rural travel, and exposure to animals or animal habitats; this information was obtained via a formal questionnaire administered to the patients and their families.

#### Pre-study period (April 2004 to April 2012)

Because the number of acute Q fever cases was relatively low compared with the CAP cases during the study period, cases of acute Q fever diagnosed before the study period were included for comparison. The same data collected during the study period were recorded retrospectively by medical chart review.

The map of case geographic distribution was made by marking the resident address of each case using SuperGIS Desktop software (Supergeo Technologies Inc.) and free vector and raster map data obtained from Natural Earth (a public domain map dataset, http://www.naturalearthdata.com).

### Statistical analyses

Categorical variables were analyzed using the Chi-square or Fisher’s exact test where appropriate. Continuous variables were analyzed using Student’s t-test. Multivariate analysis was performed using logistic regression. All *p* values were two-tailed, and a *p* value<0.05 was considered statistically significant. Data were analyzed with SPSS software for Windows (Release 15.0; SPSS, Chicago, IL).

### Ethics Statement

This study was approved by the Ethics Committee of the E-Da Hospital (EMRP-100-026). A written informed consent was obtained from enrolled patients during the study period.

## Results

### Patient collection

**Figure 1:**
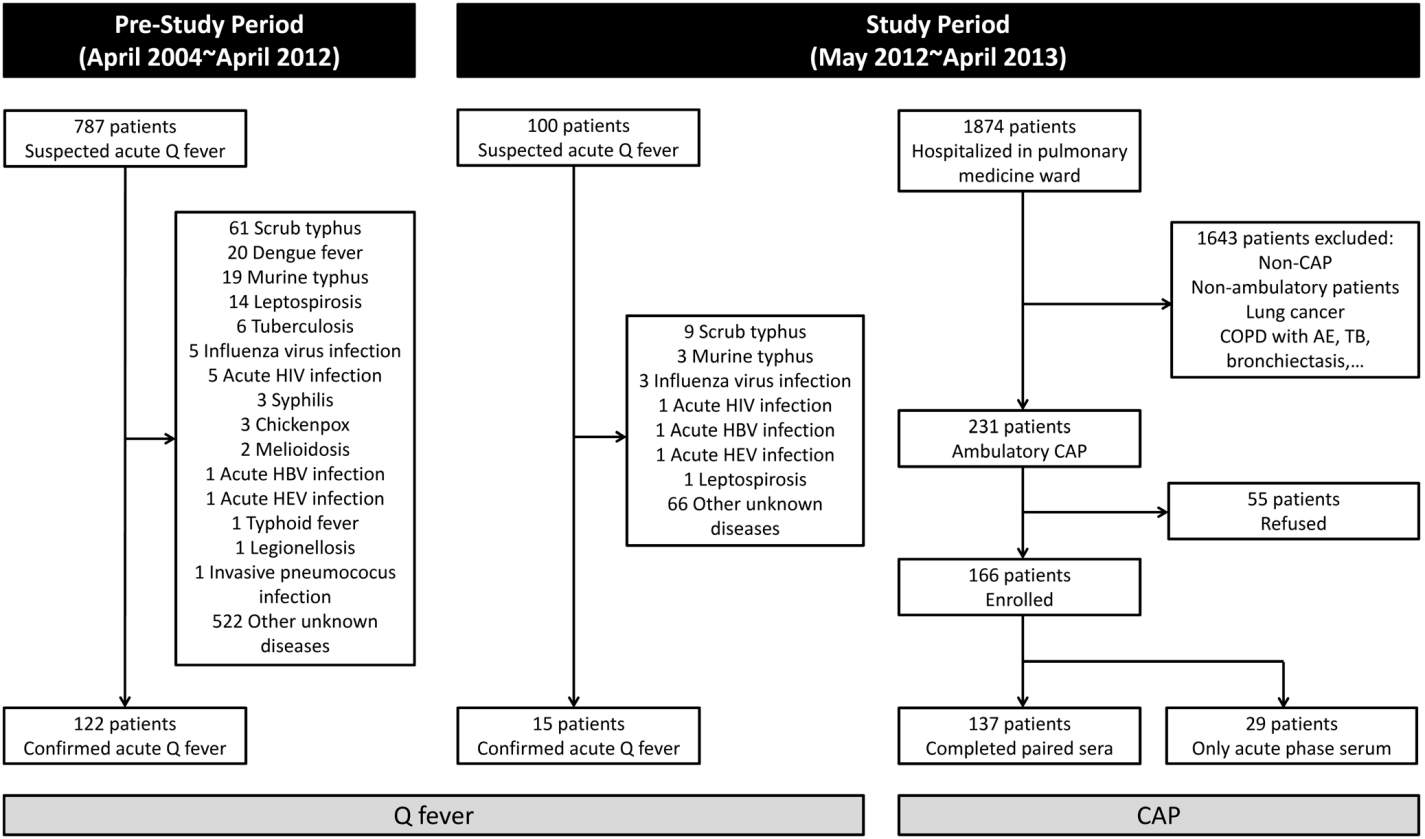
Flow chart of the recruitment of cases of community-acquired pneumonia (CAP) and Q fever.

**Figure 2:**
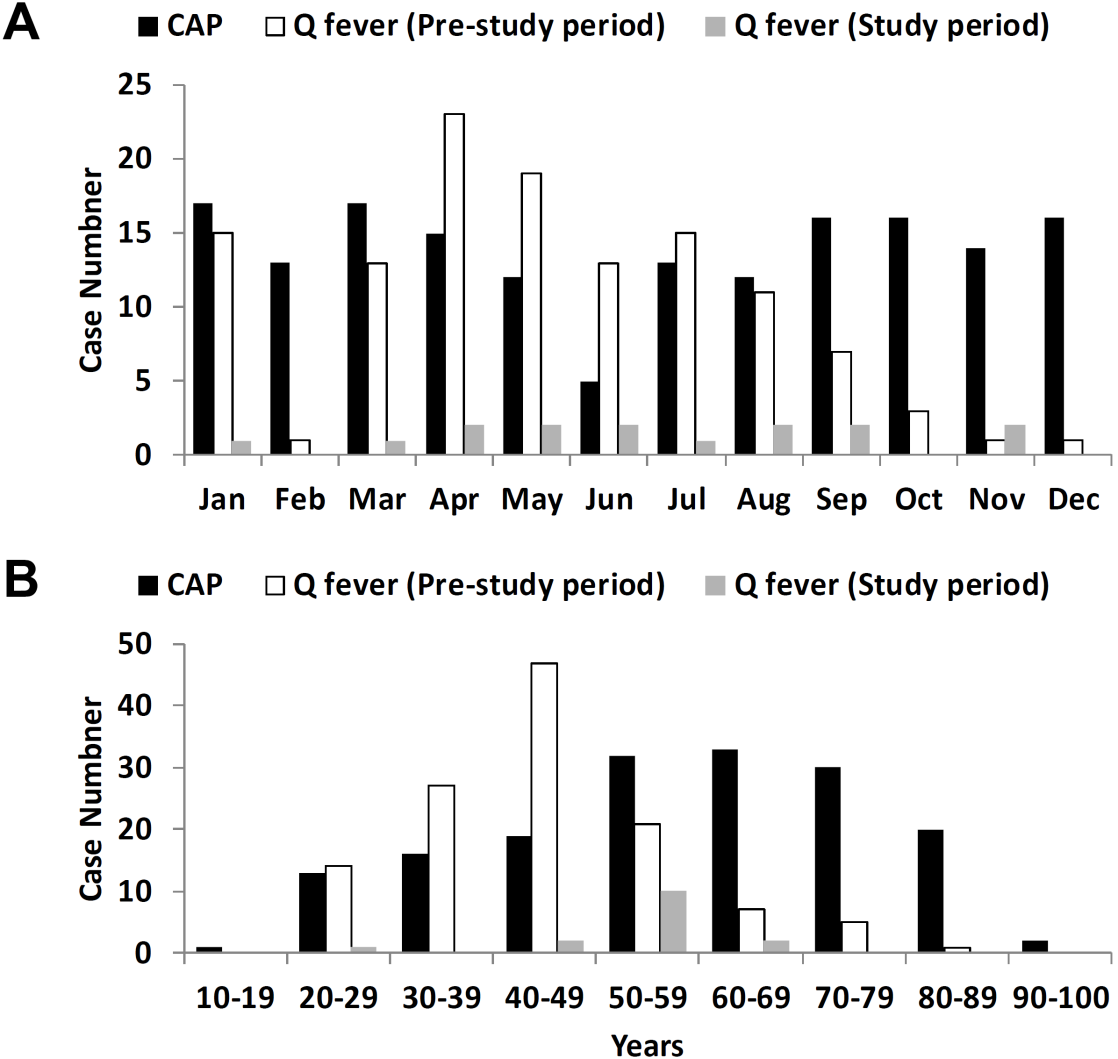
The month and age distributions of study cases. A: The month distributions of cases of community-acquired pneumonia (CAP) and Q fever. B: The age distributions of cases of CAP and Q fever.

**Figure 3:**
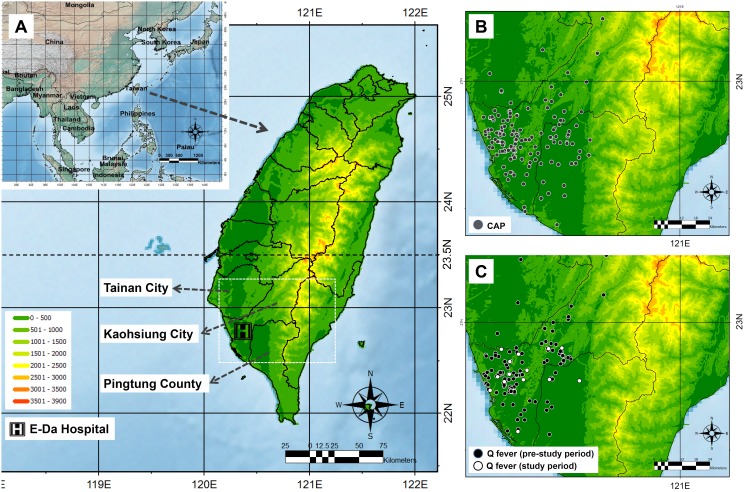
The geographic location of Taiwan and distributions of study cases. A: The geographic location of Taiwan. B: The cases’ geographic distributions for community-acquired pneumonia (CAP). C: The cases’ geographic distribution for Q fever.

The inclusion scheme for the study cases is shown in Figure 1. For CAP cases, a total of 166 cases were enrolled for study. For cases of acute Q fever, a total of 137 cases (15 and 122 cases in the study period and pre-study period, respectively) were included for comparison. The cases’ month and age distributions are shown in Figure 2A and Figure 2B, respectively. The geographic distribution of cases of Q fever and CAP was similar (Figure 3).

### The demographic characteristics and comorbidities of CAP and acute Q fever

The demographic data and underlying diseases related to CAP and acute Q fever are listed in Table 1. Comparing acute Q fever with CAP cases, acute Q fever patients were significantly younger and had a higher percentage of males and sheep/goat exposure but a lower percentage of comorbidities, including alcoholism, hypertension, diabetes mellitus, chronic obstructive pulmonary disease, and renal insufficiency, and dog or cat exposure.

**Table 1:** Demographic data, underlying diseases, and animal contact history of adult community-acquired pneumonia (CAP) and acute Q fever^a^.

Factors	CAP	Acute Q fever		
	Study period (n = 166)	Study period (n = 15)	Pre-study period (n = 122)	Total (n = 137)
Sex (male)	105 (63.3)	13 (86.7)	115 (94.3)**	128 (93.4)***
Age (mean ± SD)	58.5±17.9	52±9.6*	44.5±12.2**	45.3±12.1***
Alcoholism	39 (23.5)	4 (26.7)	11 (9.0)**	15 (10.9)***
Old pulmonary tuberculosis	6 (3.6)	1 (6.7)	0 (0)**	1 (0.7)
HBV or HCV^b^	36/166 (21.7)	2/15 (13.3)	37/121 (30.6)	39136 (28.7)
HBV	22/166 (13.3)	2/15 (13.3)	27/121 (22.3)	29/136 (21.3)
HCV	17/166 (10.2)	0/15 (0)	10/121 (8.3)	10/136 (7.4)
Liver cirrhosis	7 (4.2)	1 (6.7)	2 (1.7)	3 (2.2)
Hypertension	67 (40.4)	6 (40.0)	14 (11.5)**	20 (14.6)***
Diabetes mellitus	39 (23.5)	5 (33.3)	9 (7.4)**	14 (10.2)***
Congestive heart failure	8 (4.8)	0 (0)	2 (1.6)	2 (1.5)
Chronic obstructive pulmonary disease	23 (13.9)	0 (0)	3 (2.5)**	3 (2.2)***
Chronic renal insufficiency	21 (12.7)	0 (0)	2 (1.6)**	2 (1.5)***
Hemodialysis	4 (2.4)	0 (0)	0 (0)	0 (0)
Malignancy	9 (5.4)	0 (0)	2 (1.6)	2 (1.5)
Mountainous travel	29 (17.5)	4 (26.7)	29 (23.8)	33 (24.1)
Rural travel	51 (30.7)	5 (33.3)	39 (32.0)	44 (32.1)
Farmer	21 (12.7)	4 (26.7)	13 (10.7)	17 (12.4)
Animal husbandry	2 (1.2)	1 (6.7)	2 (1.6)	3 (2.2)
Animal Contact	144 (86.7)	9 (60.0)*	49 (40.2)**	58 (42.3)***
Dog	131 (78.9)	6 (40.0)*	25 (20.5)**	31 (22.6)***
Cat	61 (36.7)	1 (6.7)*	8 (6.6)**	9 (6.6)***
Rat	10 (6.0)	1 (6.7)	7 (5.7)	8 (5.8)
Pig	7 (4.2)	2 (13.3)	4 (3.3)	6 (4.4)
Cattle	7 (4.2)	1 (6.7)	4 (3.3)	5 (3.6)
Goat/sheep	4 (2.4)	1 (6.7)	22 (18.0)**	23 (16.8)***
Chicken	11 (6.6)	1 (6.7)	5 (4.1)	6 (4.4)
Other	8 (4.8)	2 (13.3)	7 (5.7)	9 (6.6)

SD = Standard deviation.

a Categorical variables were analyzed using the Chi-square or Fisher’s exact test where appropriate. Continuous variables were analyzed using Student’s t-test.

b Confirmed by examinations of HBsAg and anti-HCV.

*CAP vs. acute Q fever in study period, *p*<0.05.

**CAP vs. acute Q fever in pre-study period, *p*<0.05.

***CAP vs. total acute Q fever, *p*<0.05.

### The clinical manifestations of CAP and acute Q fever

The clinical manifestations of CAP and acute Q fever are listed in Table 2. Thirty-eight CAP cases (22.9%) had a CURB-65 score ≧2. Comparing acute Q fever with CAP, acute Q fever had a significantly higher percentage of cases exhibiting fever, chills, headache, jaundice, relative bradycardia, thrombocytopenia, and elevated values of liver enzymes (GPT>44 U/L or GOT>38 U/L), but a lower percentage with sore throat, cough, diarrhea, abnormal chest x-ray, leukocytosis, anemia, and serum creatinine >2.0 mg/dl.

**Table 2:** Clinical characteristics, chest x-ray, and laboratory examinations of adult community-acquired pneumonia (CAP) and acute Q fever^a^.

	CAP	Acute Q fever		
	Study period(n = 166)	Study period(n = 15)	Pre-study period(n = 122)	Total(n = 137)
Clinical symptoms and signs				
Fever	133 (80.1)	15 (100)	121 (99.2)**	136 (99.2)***
Chills	71 (42.8)	11 (73.3)*	100 (82.0)**	111 (81.0)***
Headache	70 (42.2)	7 (46.7)	88 (72.1)**	95 (69.3)***
Sore throat	51 (30.7)	0 (0)*	14 (11.5)**	14 (10.2)***
Jaundice	2 (1.2)	1 (6.7)	8 (6.6)**	9 (6.6)***
Cough	144 (86.7)	4 (26.7)*	35 (28.7)**	39 (28.5)***
Diarrhea	31 (18.7)	3 (20)	9 (7.4)**	12 (8.8)***
Arthralgia	13 (7.8)	1 (6.7)	4 (3.3)	5 (3.6)
Myalgia	73 (44.0)	7 (46.7)	39 (32.0)**	46 (33.6)
General weakness	21 (12.7)	3 (20)	14 (11.5)	17 (12.4)
Skin rash	1 (0.6)	1 (6.7)	5 (4.1)	6 (4.4)***
Relative bradycardia	22 (13.3)	5 (33.3)	57 (46.7)**	62 (45.3)***
Chest x-ray	166 (100)	15 (100)	118 (96.7)**	133 (97.1)***
Abnormal chest x-ray	166 (100)	0 (0)*	18 (15.3)**	18 (13.5)***
Unilateral infiltration	30 (18.1)	0 (0)	8 (6.8)**	8 (6)***
Bilateral Infiltration	25 (15.1)	0 (0)	10 (8.5)	10 (7.5)***
Unilateral consolidation	80 (48.2)	0 (0)*	0 (0)**	0 (0)***
Bilateral consolidation	32 (19.3)	0 (0)	0 (0)**	0 (0)***
Laboratory examinations				
Leukocytosis^b^	90 (54.2)	1 (6.7)*	5 (4.1)**	6 (4.4)***
Anemia^c^	25 (15.1)	0 (0)	0 (0)**	0 (0)***
Thrombocytopenia^d^	41 (24.7)	12 (80)*	87 (71.3)**	99 (72.3)***
White blood cell counts (/mm^3^)^e^	11761±7166	5436±2200*	6174±2521**	6093±2491***
Hemogloblin (g/dl)^e^	12.7±2.1	14.6±1.4*	14.6±1.3**	14.6±1.3***
Platelet counts (/mm^3^)^e^	206994±84985	132067±85352*	149975±124291**	148015±120523***
Creatinine (mg/dl)^e^	1.6±1.7	1.2±0.2	1.2±0.2**	1.2±0.2***
Cr>2.0 mg/dl	21 (12.7)	0 (0)	2 (1.7)**	2 (1.5)***
GPT level^e^	39.4±45.8	148.3±125.9*	130.7±77.3**	132.6±83.6***
GPT>44 U/L	40 (24.2)	11 (73.3)*	118 (97.5)**	129 (94.9)***
GPT>88 U/L	12 (7.3)	9 (60.0)*	81 (66.9)**	90 (66.2)***
GOT level^e^	54.4±86.8	153.5±124.2*	118.9±82.4**	122.3±87.4***
GOT>38 U/L	58 (36)	13 (100)*	116 (96.7)**	129 (97.0)***
GOT>76 U/L	25 (15.5)	10 (76.9)*	85 (70.8)**	95 (71.4)***
Severity of CAP (CURB-65 score)				
0	89 (53.6)			
1	39 (23.5)			
2	32 (19.3)			
3	6 (3.6)			
4 or 5	0			

GPT = Glutamate-pyruvate transaminase; GOT = Glutamate-oxaloacetate transaminase.

a Categorical variables were analyzed using the Chi-square or Fisher’s exact test where appropriate. Continuous variables were analyzed using Student’s t-test.

b Leukocytosis was defined as a white blood cell count of >10000 cells/mm^3^.

c Anemia was defined as a hemoglobin level of <10.0 g/dl.

d Thrombocytopenia was defined as a platelet count of <150000 platelets/mm^3^.

e Presented as the mean ± standard deviation.

*CAP vs. acute Q fever in study period, *p*<0.05.

**CAP vs. acute Q fever in pre-study period, *p*<0.05.

***CAP vs. total acute Q fever, *p*<0.05.

### The independent differences between CAP and acute Q fever

All characteristics that differed (i.e., with *p*<0.05) between total acute Q fever and CAP, as determined by the univariate analysis shown in Tables 1 and 2, were included in the multivariate analysis, except for the abnormal chest x-ray because it was the diagnostic criterion for CAP. Male gender (odds ratio [OR] = 22.745, 95% confident interval [CI] = 2.037∼253.958), chills (OR = 6.850, 95% CI = 1.594∼29.443), thrombocytopenia (OR = 7.254, 95% CI = 1.531∼34.370), and elevated liver enzymes (OR = 23.724, 95% CI = 2.904∼193.817) are the independent characteristics associated with Q fever, whereas alcoholism (OR = 0.083, 95% CI = 0.010∼0.654), dog contact (OR = 0.032, 95% CI = 0.006∼0.180), and cough (OR = 0.105, 95% CI = 0.021∼0.519) are the independent characteristics associated with CAP (Table 3).

**Table 3:** Significant characteristics of acute Q fever vs. adult community-acquired pneumonia by multivariate analysis.

	OR	95% CI	*p*
Male gender	22.745	2.037∼253.958	0.011
Alcoholism	0.083	0.010∼0.654	0.018
Dog contact	0.032	0.006∼0.180	<0.001
Chills	6.850	1.594∼29.443	0.010
Cough	0.105	0.021∼0.519	0.006
Thrombocytopenia^a^	7.254	1.531∼34.370	0.013
Elevated GPT or GOT level^b^	23.724	2.904∼193.817	0.003

OR = odds ratio; CI = confidence interval; GPT = Glutamate-pyruvate transaminase; GOT = Glutamate-oxaloacetate transaminase.

a Thrombocytopenia was defined as a platelet count of <150000 platelets/mm^3^.

b GPT>44 U/L or GOT>38 U/L.

### Etiology of CAP

**Figure 4:**
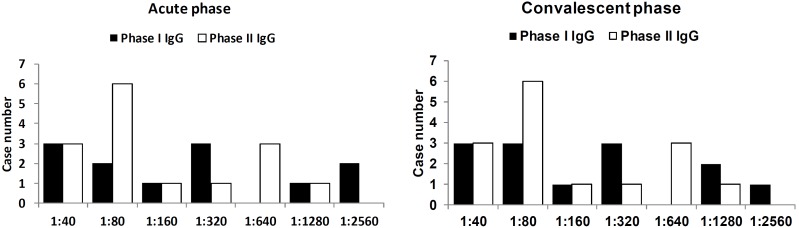
The titers of antibodies in 12 cases of community-acquired pneumonia that were seropositive for anti-phase I and anti-phase II antibodies. None had detectable IgM antibodies.

In the CAP patients, 12 (7.2%) had detectable *C. burnetii* antibodies, but none of them had seroconversion of phase II IgG or seropositivity of phase II IgM, either of which would indicate acute Q fever (Figure 4). In a total of 76 (45.8%) cases, one or more causative pathogens could be identified (Table 4). Only 58 (34.9%) cases exhibited pathogens that were identified by culture or urine antigen tests; of these, *Hemophilia parainfluenzae* (14.5%), *Streptococcus pneumoniae* (6.6%), *Pseudomonas aeruginosa* (4.8%), and *Klebsiella pneumoniae* (3.0%) were the most common. No *Legionella pneumophila* infection was found. By serology test, 15 (9.0%) cases of *M. pneumoniae*, 13 (7.8%) cases of *C. pneumoniae*, and 3 (1.8%) cases of co-infection of both pathogens were identified. It is worth noting that in 18 cases of *M. pneumoniae* or *C. pneumoniae* infections, the pathogen was not identifiable by culture or urine antigen tests alone.

**Table 4:** Bacterial etiology of adult community-acquired pneumonia (N = 166).

Bacteria identified byculture or urine antigen tests	*Mycoplasma pneumoniae*(MP), n (%)	*Chlamydophila pneumoniae*(CP), n (%)	MP andCP,n (%)	Not MP or CP, n (%)
No identifiable pathogen by culture or urineantigen tests (n = 108, 65.1%)	7 (4.2)	10 (6.0)	1 (0.6)	90 (54.2)
Mixed normal flora (n = 69, 41.6%)	5 (3.0)	6 (3.6)	1 (0.6)	57 (34.3)
No culture available (n = 39, 23.5%)	2 (1.2)	4 (2.4)		33 (19.9)
Bacteria identified by culture or urineantigen tests (n = 58, 34.9%)	8 (4.8)	3 (1.8)	2 (1.2)	45 (27.1)
Gram positive bacteria (n = 10, 6.0%)				
* Streptococcus pneumoniae* (n = 9, 5.4%)^a^	1 (0.6)			8 (4.8)
* Staphylococcus aureus* (n = 1, 0.6%)				1 (0.6)
Gram negative bacteria (n = 43, 25.9%)				
* Haemophilus parainfluenzae* (n = 23, 13.9%)^b^	4 (2.4)	1 (0.6)		18 (10.8)
* Pseudomonas aeruginosa* (n = 5, 3.0%)^c^	1 (0.6)		1 (0.6)	3 (1.8)
* Klebsiella pneumoniae* (n = 4, 2.4%)^d^	1 (0.6)		1 (0.6)	2 (1.2)
* Escherichia coli* (n = 3, 1.8%)				3 (1.8)
* Haemophilus influenzae* (n = 2, 1.2%)^e^				2 (1.2)
* Acinetobacter baumannii* (n = 2, 1.2%)^f^		1 (0.6)		1 (0.6)
* Acinetobacter lwoffii* (n = 1, 0.6%)				1 (0.6)
* Enterobacter cloacae* (n = 1, 0.6%)^g^				1 (0.6)
* Enterobacter aerogenes* (n = 1, 0.6%)				1 (0.6)
* Eikenella corrodens* (n = 1, 0.6%)				1 (0.6)
Mixed Bacterial infection (n = 5, 3.0%)				
* Acinetobacter baumannii* + *Pseudomonas* *aeruginosa* (n = 1, 0.6%)		1 (0.6)		
* Streptococcus pneumoniae* + *Pseudomonas* *aeruginosa* (n = 1, 0.6%)				1 (0.6)
* Streptococcus pneumoniae* + *Haemophilus* *parainfluenzae* (n = 1, 0.6%)				1 (0.6)
* Haemophilus influenza* + *Citrobacter* *freundii* (n = 1, 0.6%)				1 (0.6)
* Pseudomonas aeruginosa* + *Klebsiella* *pneumoniae* + *Enterobacter* *cloacae* (n = 1, 0.6%)	1 (0.6)			
Total (n = 166, 100%)	15/166 (9.0)	13/166 (7.8)	3/166 (1.8)	135 (81.3)

a *Streptococcus pneumoniae* accounts for 6.6% (11/166).

b *Haemophilus parainfluenzae* accounts for 14.5% (24/166).

c *Pseudomonas aeruginosa* accounts for 4.8% (8/166).

d *Klebsiella pneumoniae* accounts for 3.0% (5/166).

e *Haemophilus influenzae* accounts for 1.8% (3/166).

f *Acinetobacter baumannii* accounts for 1.8% (3/166).

g *Enterobacter cloacae* accounts for 1.2% (2/166).

### Endemic data on Q fever during the study period in Taiwan

During the study period, a total of 55 cases of acute Q fever were confirmed by the Taiwan CDC, and 22 cases were distributed in Kaohsiung City [48]. Among them, 15 cases were identified from E-Da hospital, and none presented as pneumonia.

## Discussion

The clinical characteristics of Q fever are poorly described in tropical regions. We demonstrate that hepatitis is the major presentation of Q fever and that Q fever is not identified in CAP during the study period in southern Taiwan. In contrast, pneumonia is the major presentation of Q fever, and 24.4% of CAP is attributable to Q fever in French Guiana [21]. In Thailand, among the 9 cases of Q fever identified in 678 patients with acute febrile illness, 2 and 3 presented with pneumonia and hepatitis, respectively [23]. A recent systematic review regarding Q fever in Africa revealed that the human seroprevalence was <8% and that Q fever accounted for 2–9% of febrile illness hospitalizations [49]. Moreover, new reports revealed a high seroprevalence of Q fever in tropical regions of Africa [24]–[27]. In West Africa, the seroprevalence could be as high as 24.5% and 27–45% in Senegal [24] and Togo [25], respectively. In East Africa, Q fever accounts for 5.0% of hospitalized patients with non-malaria febrile illness in Tanzania [26], and in Kenya, the seroprevalence is 30.9%, and it accounts for 3% of patients with acute lower respiratory illness [27]. However, no clinical characteristics were available from these reports. The high seroprevalence without reports of clinical cases of Q fever suggested that Q fever is a neglected disease in tropical regions. The relatively limited medical resources, paucity of diagnostic tools, and preconceived diagnosis of tropical febrile diseases, such as malaria and dengue fever, might cause Q fever to be overlooked in the tropics.

This is the first study that provides evidence that Q fever is not a common etiological agent of CAP, and the major presentation of Q fever is hepatitis rather than pneumonia in Taiwan. The exact cause of the geographic variation in the presentation of acute Q fever is not clear, and several possible reasons are supposed, including selection bias by specialists and differences in case definition [2], host factors [50], [51], bacterial strains [51], and transmission routes [52], [53]. The possibility of selection bias in retrospective clinical case studies and the diagnostic habits of clinicians were excluded because all of the CAP patients were tested for Q fever and because almost all Q fever cases had chest x-rays examined in this study. Because the ingestion of raw milk is an uncommon habit in Taiwan, the dominance of hepatitis does not seem to be attributable to this transmission route. Geographic differences in the presentation of Q fever also exist in Eastern Asia. Pneumonia is the predominant presentation of Q fever in Japan, particularly for children with atypical pneumonia [15]. In South Korea, however, only 10.7% of cases present with pneumonia, and 21.4% are hepatitis [16]. The presentation of Q fever in China is not well illuminated in English literature. In the present study, including 137 cases of Q fever, nearly 95% of cases had elevated liver enzymes, and only 13.5% had abnormalities in chest x-ray (Table 2). Similarly, one report from Hong Kong, a region near Taiwan, revealed that all of the 8 identified cases had liver dysfunction and only 2 had pulmonary involvement [54]. Accordingly, we speculate that bacterial strain or host factors might be the cause of predominance of hepatitis over pneumonia in Q fever in Taiwan.

In addition to a lower rate of pneumonia and respiratory tract symptoms (i.e., cough and sore throat) in Q fever, several differences were found between Q fever and CAP cases via univariate analysis (Tables 1 and 2). In general, Q fever has a higher percentage of males, a younger mean age, fewer underlying chronic diseases, more symptoms and signs of fever, chills, and headache, relative bradycardia, and a higher percentage of normal leukocyte counts, thrombocytopenia, and elevated liver enzymes. Having a high-risk occupation and animal exposure are the most considerable risk factors emphasized for clinical suspicion of Q fever; however, the percentages of farmers and animal husbandry workers are low in both Q fever and CAP, and no significant difference exists between them. Regarding animal exposure, overall, patients with Q fever have a significantly higher rate of goat/sheet exposure than patients with CAP (16.8% vs. 2.4%, *p*<0.05); however, the difference is not significant during the prospective study period (6.7% vs. 2.4%, *p*>0.05) (Table 1). Low-risk occupations and goat/sheep exposure rates could be explained by the inhalation of infectious particles spreading from a remote source in an endemic area. Based on the multivariate analysis, male gender, chills, thrombocytopenia, and elevated liver enzymes are more common in Q fever, and alcoholism, dog contact, and cough are more common in CAP. These results suggested that clinical characteristics might be more useful than the risk category of the patient’s occupation and goat/sheep exposure for determining clinical suspicion of Q fever. Men are the primary workers and participants in outdoor activities in Taiwan, and this increases the possibility of exposure to the infectious organisms. In addition, the protective effect of female hormones in *C. burnetii* infection demonstrated in animal studies might partially explain the male predominance of Q fever [55]. In the study by Raoult et al., thrombocytopenia is significantly more frequent in acute Q fever patients who present with hepatitis compared to those with pneumonia or isolated fever [51]. In addition, the presence of antiphospholipid antibodies in patients with Q fever who presented with hepatitis might partially explain the high frequency of thrombocytopenia [1], [56], [57]. Alcohol abuse could alter human immunity and increase susceptibility to bacterial pneumonia, which may explain the higher percentage of alcoholism in CAP [58]. Dogs are common as pets and stray animals, which might be easily missed when taking a patient history regarding Q fever that focuses on the main reservoir animals, such as sheep, goats and cattle. These could be valuable characteristics for distinguishing Q fever from CAP, particularly in regions where hepatitis is the major presentation of Q fever.

Although no acute Q fever was found in CAP, we identified the seroprevalence of Q fever in CAP to be 7.2%. This was a little higher than in other population in southern Taiwan in which the seroprevalence rate was 4.2% in both hospitalized patients and in physical examination participants [59] and 2.7% in serum samples obtained from a dengue fever investigation but much lower than the veterinary-associated populations (26.3%) [60]. In Canada, pneumonia and hepatitis are the predominant presentations of Q fever in Nova Scotia [4] and Ontario [11], respectively. In the multi-province CAP study conducted by Marrie et al., Q fever accounted for 0.9% of CAP, and the overall seroprevalence was 13.8% [34]. In addition, the seroprevalence and percentage of Q fever in CAP seems not to differ between Nova Scotia (12.2% and 0.7% [1/145]) and Ontario (10.8% and 0.5% [1/193]). These findings indicated that even in an area in which Q fever predominantly presents with hepatitis, there is a significant seropositive rate for Q fever among CAP patients, similar to the results found in our study. Of note, 3 patients had an anti-phase I IgG titer ≧1280, suggesting chronic Q fever (Figure 4). However, no *C. burnetii* DNA was detectable in their serum or peripheral blood monocytes by PCR examination [47], and the diagnosis of chronic Q fever could not be confirmed.

During the study period, 15 cases of acute Q fever were identified, which accounted for about two thirds and one fourth of the cases in Kaohsiung City (22 cases) and Taiwan (55 cases), respectively. Kaohsiung City is the region in which the most Q fever cases were confirmed in Taiwan (Figure 3). This indicated that Q fever is still endemic during the study period and that despite these results coming from a single study center, the number of identified cases of Q fever may be representative of the situation in Taiwan. We previously reported that 49.5% of acute Q fever was accompanied by seropositivity for *M. pneumoniae* IgM based on an examination using ELISA kits (Savyon Diagnostics, Ashdod, Israel) in this study and speculated that this might result from the cross-reactivity of Q fever serum with the ELISA kits. We were therefore concerned that Q fever might be misdiagnosed as *M. pneumoniae* infection [41]. In this study, however, no acute Q fever was identified in CAP cases, not even in the 31 cases of *M. pneumoniae* or *C. pneumoniae* infection. However, in 12 of the 15 Q fever cases that had paired serum available for examination, 0 (0%) and 4 (33.3%) cases were seropositive for *M. pneumoniae* IgM based on results from the ELISA kits used in this study and others (NovaTec Immundiagnostica GmbH, Germany), respectively (data not shown). These results supported our previous speculation that acute Q fever serum has cross-reactivity with ELISA kits of *M. pneumoniae* IgM, but no *M. pneumoniae* infection was misdiagnosed as acute Q fever by IFA tests.

In clinical settings, cultures of respiratory specimens or blood and urine antigen tests are the most common methods used to identify the etiology of pneumonia. In our study, however, the causative pathogen could be identified in only 34.9% of cases by these methods. In the remaining 65.1% of cases, 23.5% had no sputum available, and 41.6% had poor quality sputum samples for culture despite the diagnosis of pneumonia. By using serological examinations, an additional 18 (10.8%) cases of *M. pneumoniae* or *C. pneumoniae* infections could be identified, and the overall identification rate increased to 45.8% (Table 4). The identification rate is slightly lower than those found in previous prospective CAP studies in Taiwan with 58.9% [42] and 72% [43]. This is most likely because *M. tuberculosis* was excluded and testing for virus was not part of the examination. The identification rate of the causative pathogens of CAP varied from approximately 30% to 85%, depending on the study design, population included, and examination methods of pathogen identification. *S. pneumoniae*, *M. pneumoniae*, *C. pneumoniae*, and Q fever account for 5.6–85%, 0.7–61.3%, 0.1–11%, and 0.3–24%, respectively [18], [21], [29], [30], [33], [35]–[37], [61], [62]. However, the prevalence of *S. pneumoniae* is 6.6%, *M. pneumoniae* is 9.0%, and *C. pneumoniae* is 7.8%, but no Q fever was identified in this study. Despite the fact that all included cases had a urine *S. pneumoniae* antigen test, the percentage of *S. pneumoniae* is lower than that in the literature. This might be because respiratory specimens were not collected consistently with regard to either quantity (i.e., 23.5% of cases had no specimen) or quality (i.e., 41.6% of culture results showed normal flora). This indicated that without aggressive diagnostic approaches and the strict selection of respiratory specimens, the etiology of pneumonia is difficult to identify in clinical practice.

There are several limitations in this study. The one year study period might be short, but this is the longest prospective study of CAP and Q fever in Taiwan. Although this is a single unit study, it accounted for nearly one fourth of Q fever cases identified in Taiwan. The data on Q fever cases during the pre-study period were recorded retrospectively, but few differences were found compared with those recorded prospectively in the study period. Virus was not included, and the collection of qualified respiratory specimens for culture was not implemented strictly for the survey of CAP etiology. However, this situation accurately reflected the cost considerations and imperfect practices in clinical settings.

In conclusion, Q fever is not a common etiology of CAP in hospitalized and ambulatory adults in a tropical region of southern Taiwan. The major presentation of Q fever is fever with hepatitis rather than pneumonia. The identification rate of the etiology of CAP is low by routine clinical practice.
